# Statistics of Sudden Deaths, More Particularly of Deaths from Apoplexy, in the City and Neighbourhood of Milan, from 1750 to 1834

**Published:** 1840-10

**Authors:** 


### Art. VI.

1. *Statistica delle Morti Improvvise, e particolarmente delle Morti per Appoplessia, nella Citta e nel Circondario Esterno di Milano, dall' anno* 1750 *al* 1834. Del Giuseppe Ferrario, d.m.—*Milano,* 1834. 8vo, pp. 238.

*Statistics of Sudden Deaths, more particularly of Deaths from Apoplexy, in the city and neighbourhood of Milan, from* 1750 *to* 1834. By Joseph Ferrario, m.d.—*Milan,* 1834.

2. *Monografia sulle Morti Repentine.* Del Signor N. M. Sormani, d.m. —*Milano,* 1834.

*Monography on Sudden Deaths.* By N. M. Sormani, m.d.—*Milan,* 1834.

The two works of the Doctors Ferrario and Sormani, at the head of this article, are two Prize Essays upon the following theme, proposed by the Lombardo-Venetian Royal Institute of Science, Literature, and Arts:—"To investigate the causes of sudden death; determine the morbid alterations by which it is produced; the influence of the various constitutions of the atmosphere, of the seasons, of meat and drink, of the passions, the manner of life, &c.; to enquire, also, whether this kind of death is become more common in the present day than it was formerly."

Both the essays are very creditable productions; and the writers examine the subject from different points of view. Dr. Ferrario has entered into an elaborate investigation of the medical topography of Milan; and we shall lay some of the principal results of his labours before our readers, as it may be interesting to compare the cities of Italy with the cities of this country, where the sky is imagined to be less genial. Milan, in its sanatory state, represents with sufficient accuracy the cities of the north of Italy; except that it appears to have been some fifty years ago rather more salubrious than Lodi, Cremona, Mantua, and Pavia. The annual deaths in 1790 were, in Milan, 1 in $28\frac{2}{3}$, Como, 1 in $29\frac{1}{2}$, Lodi, 1 in $19\frac{3}{4}$, Pavia, 1 in $25\frac{1}{2}$, Cremona, 1 in 19, Mantua, 1 in $22\frac{2}{3}$, Casal Maggiore, 1 in $20\frac{1}{2}$, Varese, 1 in $30\frac{1}{3}$. The mortality in all the cities was 1 in 26; while in the surrounding country, the *campagna* of the several cities, the mortality was rather higher, 1 in 24. This is in contradiction with nearly all other observations; but Ferrario accounts for it by the greater humidity of the country, and the practice of sending infants, particularly foundlings, out of the cities to be suckled. In the three years 1831-2-3, the population of Lombardy increased from 2,393,424 to 2,416,567, and the annual proportion of deaths in the three years respectively was 1 in 30, 1 in 28, 1 in 28; of births, 1 in 24, 1 in 26, 1 in 25.

Milan, the capital of Lombardy, had, in 1833, a population of 179,625; namely, 155,472 in the city, and 24,153 in the *Corpi Santi,* or suburbs, exclusive of the garrison. The city itself is 12,749 metres in circumference; and the area is computed at 8,683,450 English square yards. It lies in a fertile level plain, between the rivers Adda and Ticino. The inhabitants, according to Ferrario, are goodnatured, frank, and generous; in proof of which he alleges the various institutions for the relief of the poor, who are, not as elsewhere, acquainted with real destitution (*vera miseria.*) The city contains 4920 houses, generally of three stories, besides the ground-floor; several, however, have four, five, and even six stories. There are consequently 32 persons to each house; and 1 person to every 56 square yards of area. The soil is alluvial; and potable water is obtained from wells in every house. The Botanic Garden stands 121 metres above the Adriatic. The latitude of Milan is 45° N.; and the longest summer day is 15 hours 34 minutes; the shortest day of winter is 8 hours 36 minutes. The mean height of the thermometer, deduced from observations made twice a day during 55 years by the eminent astronomer Cæsaris was 60° Fahrenheit: the mean of all the maxima was 88°, of all the minima 19°. During the 71 years, 1763 to 1833, the north wind (*settentrionale*) blew 2321 days, the north-east (*Greco*) 3614 days, the east (*Levante*) 6600 days, the south-east (*Sirocco*) 2357½ days, the south (*Australe*) 1506½ days, the south-west (*Libeccio*) 3339½ days, the west (*Ponente*) 5083 days, the north-west (*Maestro*) 2352 days. The annual fall of rain is 35 inches.

Milan is said to have contained 246,000 souls in 1590; and in the history of Milan, by Verni, the pestilence of 1630 is said to have proved fatal to 140,000 citizens. The population is given in the tables of the census, by Count Carli, as 110,118 in the year 1750; and the deaths then amounted to 2023, exclusive of the deaths in hospital. The *Spedale Maggiore* is an immense establishment: it contained 1067 patients on the 1st of January 1780, and 1334 on the 1st of January 1833. The total number of patients admitted in the 54 years, 1780 to 1833, was 1,063,978; of whom 146,219 or 13·76 per cent. died within the walls. The mortality of cases treated in 1780-800 was 13·97 per cent.; in 1801-33 a little less or 13·60 per cent. In one year 16 out of 100 cases terminated fatally; in several other years not more than 12 out of 100. The total deaths in Milan in the 54 years, 1780-833, amounted to 373,256, and the deaths in the *Spedale Maggiore* were 146,219, or 39 per cent. of the whole number. In the year 1832 the total deaths in Milan and the suburbs were 7405; namely, 2916 in the great hospital, 65 in the lunatic asylum of Senavra, 99 in the hospital of Fatebenefratelli, and 409 in the foundling hospital (*Pia Casa di S. Caterina alla ruota*); for, like other cities on the continent, Milan has its foundling hospital—that bounty upon profligacy, parental depravity, and indirect infanticide. The number of parents who abandon their children progressively increased from 592 in 1750, to 2792 in 1833. In 1821-33 there were 87,484 children born in Milan; and of these 30,418, or 35 per cent. were abandoned. In the 3 years, 1831-3, 18,121 children were born in the city of Milan, and 4314, 23·8 per cent., died in the first year of life.

From an enumeration made under the direction of the celebrated Gioja, in 1805, it appears that there were then in Milan 100 physicians, 123 surgeons, 49 accoucheurs, 125 chemists, druggists, &c. (*chimici e farmacisti, padroni e praticanti.*) The population was 150,442.

In order to deduce the rate of mortality of any place it is necessary to ascertain the number of deaths during a certain number of years, and the mean number living during the same period. The number living is ascertained by regular enumerations; the deaths by the bills of mortality. But in all cities where there are large hospitals the sick are brought from the country; and these ought to be excluded from the calculation. Again, many of the children of foundling hospitals are sent into the country; and these should by right be included in the enumeration of the living, and, when they die, in the bills of mortality. Thus the average number of children in St. Catherine's hospital (1833) was 342, the deaths in the hospital 705; the average number of foundlings in the country was 6484; the deaths among them in the year, 745, or 11·5 per cent. The two elements act in opposite directions, and neutralize each other to a certain extent. In every city there is a *floating population* (*popolazione mobile*) consisting of the entire number of persons living within the limits, and a *fixed population* (*popolazione stabile*), comprising none but permanent residents, or citizens. The fixed population in Milan (1833) was 136,508, namely, 68,203 males, and 68,305 females: the fixed and floating population was 179,625; the difference was 43,117; and in the same year 2437 are stated to have died in the hospitals, not belonging to Milan (*non appartenenti alla città di Milano.*)

The subjoined table exhibits the mortality of the population of Milan during 80 years, in decennial periods. We have deduced these results from the 57 enumerations of the population, and from the annual deaths given by Ferrario.

*Mortality of Milan*, 1751-1830.

| Years. | Censuses. | Mean Population. | Annual Deaths. | Annual Deaths per cent. |
|---|---|---|---|---|
| 1751-60 | 2 | 117390 | 5521·4 | 4·703 |
| 1761-70 | 9 | 128074 | 5018·6 | 3·918 |
| 1771-80 | 10 | 134249 | 5471·5 | 4·076 |
| 1781-90 | 10 | 132795 | 6153·2 | 4·634 |
| 1791-800 | 8 | 134074 | 7174·2 | 5·339 |
| 1801-10 | 4 | 146148 | 7223·0 | 4·942 |
| 1811-20 | 4 | 156596 | 7180·0 | 4·585 |
| 1821-30 | 10 | 170695 | 6852·1 | 4·021 |
| | 57 | 140003 | 6324·5 | 4·517 |

Some slight deduction should probably be made for the sick sent to the hospital from the surrounding country.

Milan was occupied by the French army and revolutionized in 1796; the new government was broken up by the arrival of the Austrian and Russian armies in April, 1799; and the French regained possession of the city in June, 1800. It is not often that we have so good an opportunity of ascertaining the effect of war upon the mortality.

| Annual Mortality per cent. | | | | Births to 100 living. | Mean Price of grain. |
|---|---|---|---|---|---|
| Years. | Males. | Females. | Mean. | | |
| | 4·961 | 4·524 | 4·742 | 4·094 | 28·87 lire |
| War ... 1796-805 | 5·859 | 5·354 | 5·606 | 4·474 | 44·51 |

The annual mean price of grain remained nearly stationary at 33 *lire al moggio* from 1793 to 1798; it had been as low as 21·83 lire in 1791; but in 1799 it rose to 38·35, in 1800 to 58·43, in 1801 to 67·09 lire: in 1833 it was 33·35 lire. It will be observed that when the annual mortality increased from 4·742 per cent. to 5·606, or 18 per cent. the births increased 9 per cent., or in half the proportion, so that an effort was made by nature to replace the excessive loss by excessive reproduction.

Dr. Ferrario has endeavoured to determine the influence of the three systems of physic which prevailed in Milan during the 84 years, 1750 to 1833; and it may not be uninteresting to compare the mortality in the three epochs, although it would be unfair to ascribe the differences exclusively to the medical systems.

| Years. | Annual Mortality. | Observations. |
|---|---|---|
| 1750-9 | 1 in 22·08 | Systems of Boerbave, Cullen, Tissot, Borsieri, &c. Epidemics of smallpox at intervals, and often very severe. |
| 1760-9 | 1 in 24·44 | |
| 1770-9 | 1 in 24·55 | |
| 1780-9 | 1 in 22·31 | System of Brown. Period of war and revolutions. |
| 1790-9 | 1 in 19·12 | |
| 1800-8 | 1 in 17·05 | Introduction of vaccination. The system of contrastimulism commences and is in vogue. Changes of government. Petechial fever. |
| 1812-19 | 1 in 17·18 | |
| 1820-9 | 1 in 25·18 | Period of tranquillity. The doctrine of contrastimulism is moderated by eminent teachers, particularly by Hildebrandt. |
| 1830-3 | 1 in 25·29 | |

It would be absurd to pretend that medical theories have not a decided influence upon the mortality; but it is difficult to say, in the present case, how far contrastimulism is chargeable with the excessive mortality in 1800-19.

We now come to the more direct subject of the essay—the causes and prevalence of sudden deaths. What is sudden death?—"That," says Morgagni, "which, foreseen or not, takes away a man's life suddenly, without any expectation of the event upon his part." It may be asked does "suddenly" mean three minutes, three hours, or three days? and there can be no doubt that in this as in other cases the duration of the disease should be stated definitely; nevertheless, in the absence of more accurate facts, the general notion of sudden death is not to be discarded as valueless. It appears to comprehend a class of cases generally terminating in less than three days after the attack. Sormani has given a good practical classification of the causes of sudden death, which we subjoin.

"*Causes of Sudden Death.*

A. EXTERNAL.

(*a*) *Known.* 1. From a total and sudden privation of respirable gas—drowning—suffocation, &c.

2. Poisons, particularly hydrocyanic acid.

3. Lightning.

4. Traumatic lesions—wounds of the great vital centres—cerebro-spinal concussion—contusions and extensive lacerations, &c.

5. Excesses of heat, cold, meat, drink.

(*b*) *Unknown.* 1. Certain deleterious states of the atmosphere entirely unknown.

2. Miasmatic emanations from animal and vegetable matter in putrefaction.

3. Pestilential contagions of several kinds.

4. Poison of the viper, virus of hydrophobia.

B. INTERNAL (or internal morbid states).

(*a*) *That can be detected in the Body.* Foreseen during life (1) by symptoms of the hemorrhagic, apoplectic, cardiopathic, or pulmonic habit; (2) by symptoms of diseases which often destroy life suddenly: vertigo, cerebral, and pulmonary apoplexy, epilepsy, hydropericardium, asthma, or syncope from organic disease of the heart, angina pectoris, &c.

*Unforeseen during life*, (1) as they commence insidiously and gradually increase; several of the diseases previously mentioned, sometimes unattended by their peculiar symptoms; (2) as the sudden death is caused more by the intervention of an external cause than by the maturity of the internal pathological conditions: e. g., the tenuity of the parietes of a blood-vessel might have resisted secretly the impulse of the blood, driven there by the heart, had not a heavy fall, or a paroxysm of anger, made the impulse greater for a moment, and ruptured the vessel in the diseased part.

(*b*) *That cannot be detected in the Body;* (1) because they arise in the intimate tissue of vital organs, or consist of an altered state of the blood, miasmatic fevers, malignant fevers; or (2) because they act upon the innervation with the rapidity and force of lightning, without leaving any traces which our senses or the finest anatomical instruments can discover; nervous apoplexy from an internal cause, violent mental affections, sometimes with the coincidence of physical disorders."

Dr. Sormani states that out of 22,452 deaths at Milan in the three years 1831-3, there were 875 by apoplexy, 25 by hemorrhage, 1 by pulmonary apoplexy, 11 by syncope and asphyxia, 5 by angina pectoris, 29 by aneurism, 91 by diseases of the heart, 1 by cerebral concussion, 2 by vertigo, 1 by coma, 2 by suffocative catarrh, 5 by difficult labours; in all 1048, which would frequently fall under the designation of sudden death.

In his statistical researches Ferrario has restricted himself to the investigation of apoplexy, or the diseases designated apoplexy in the bills of mortality. Assuming that the term apoplexy has been employed in the same sense, mortality from that disease has doubled in Milan during the period comprised in the bills of 1750 to 1833; while the mortality from other diseases has not decreased to the same extent.

*Proportion of Annual Deaths from Apoplexy.*

| Years. | Deaths by Apoplexy. | Proportion of the Deaths by Apoplexy to the Population. |
|---|---|---|
| 1750-9 | 959 | 1 to 1231 |
| 1760-9 | 976 | „ 1296 |
| 1770-9 | 1103 | „ 1205 |
| 1780-9 | 1235 | „ 1092 |
| 1790-9 | 1718 | „ 766 |
| 1800-8 | 1679 | „ 847 |
| 1812-9 | 1665 | „ 965 |
| 1820-9 | 2267 | „ 743 |
| 1830-3 | 1209 | „ 589 |
| 1750-1833 | 12811 | „ 917 |

It is found that males are more liable than females to apoplexy. Of 11,731 cases of apoplexy in Milan, 6492 were of males, and 5239 of females; while the proportion of females living is higher than that of males.

A greater number of cases of apoplexy occurred in the cold than in the hot months. The deaths from apoplexy in January were 1176, February 1030, March 956, April 848, May 829, June 681, July 681, August 645, September 718, October 822, November 963, December 1075—total, 10,432.

It is not easy to determine the influence of professions; but the following is an estimate given by Ferrario.

*Deaths by Apoplexy out of* 100,000 *of each Class.*

| | |
|---|---|
| Brokers, Agents, Farmers (*Sensali, Agenti, Affettajuoli*) | 1,117 |
| Physicians, Surgeons | 480 |
| Painters, Engravers, Sculptors | 329 |
| Merchants | 256 |
| Victuallers and Innkeepers, Winehouse-keepers, Cheesemongers (*Osti, bettolieri, pizzicagnoli*) | 255 |
| Engineers and Accountants (*Ingegneri e ragionieri*) | 168 |
| Masons | 32 |

The most important result is that deduced from the deaths by apoplexy at different ages. The tendency to apoplexy increases with age in a geometrical progression; doubling every 10 years. The ages of the living were enumerated in 1805; the deaths in decimal periods of life in 57 years (1774 to 1833, excepting 3 years) are given in the following table by Ferrario. The right-hand column shows the mortality from apoplexy, upon the assumption that the ratio was uniform—or 2·15 per cent. every 10 years.

*Table of the Living in* 1805; *the Total Deaths in* 1805; *the Deaths by Apoplexy,* 1774-1833; *and the Annual Deaths per cent. by Apoplexy.*

| Age. | Living, 1805. | Total Dying, 1805. | Died of Apoplexy, 1774-1833. | Annual Mort. per cent. by Apoplexy. (Observed.) | (Calculated.) |
|---|---|---|---|---|---|
| 0-10 | 35837 | 2842 | 147 | ·007 | ·006 |
| 10-20 | 26555 | 264 | 221 | ·015 | ·013 |
| 20-30 | 25639 | 363 | 407 | ·028 | ·029 |
| 30-40 | 22010 | 335 | 779 | ·062 | ·062 |
| 40-50 | 17733 | 401 | 1361 | ·135 | ·132 |
| 50-60 | 11704 | 403 | 2151 | ·322 | ·285 |
| 60-70 | 7768 | 454 | 2849 | ·643 | ·614 |
| 70-80 | 2699 | 358 | 2033 | 1·321 | 1·321 |
| 80-90 | 474 | 171 | 461 | 1·706 | |
| 90-100 | 23 | 12 | 23 | | |
| | 150442 | *5603 | 10432 | ·122 | |

Ferrario has given the total number of deaths at different ages only for the year 1805. And although he has given the mortality at every age by apoplexy in vulgar fractions, he does not appear to have been aware that the rate of mortality increased regularly, or at least so uniformly as to leave little doubt that the fatality of apoplexy is governed by a mathematical law. His monograph, however, contains a valuable collection of facts, and throws considerable light upon the etiology of apoplexy.

### Art. VII.

* The total deaths in 1805 amounted to 6577; but the number 5603 is exclusive of foreigners, and of deaths of persons in the hospitals not belonging to the city of Milan.

